# The Role of Structure and Interactions in Thermoplastic Starch–Nanocellulose Composites

**DOI:** 10.3390/polym13183186

**Published:** 2021-09-20

**Authors:** Emília Csiszár, Dávid Kun, Erika Fekete

**Affiliations:** 1 Laboratory of Plastics and Rubber Technology, Department of Physical Chemistry and Materials Science, Budapest University of Technology and Economics, Műegyetem rkp. 3, H-1111 Budapest, Hungary; csiszar.emilia@vbk.bme.hu (E.C.); bodine.fekete.erika@vbk.bme.hu (E.F.); 2 Research Centre for Natural Sciences, Institute of Materials and Environmental Chemistry, Magyar Tudósok Körútja 2, H-1117 Budapest, Hungary

**Keywords:** thermoplastic starch, crystalline nanocellulose, plasticizers, composite film, mechanical properties, morphology, reinforcement, competitive interactions, aging, retrogradation

## Abstract

Composite films were fabricated by using cellulose nanocrystals (CNCs) as reinforcement up to 50 wt% in thermoplastic starch (TPS). Structure and interactions were modified by using different types (glycerol and sorbitol) and different amounts (30 and 40%) of plasticizers. The structure of the composites was characterized by visible spectroscopy, Haze index measurements, and scanning electron microscopy. Tensile properties were determined by tensile testing, and the effect of CNC content on vapor permeability was investigated. Although all composite films are transparent and can hardly be distinguished by human eyes, the addition of CNCs somewhat decreases the transmittance of the films. This can be related to the increased light scattering of the films, which is caused by the aggregation of nanocrystals, leading to the formation of micron-sized particles. Nevertheless, strength is enhanced by CNCs, mostly in the composite series prepared with 30% sorbitol. Additionally, the relatively high water vapor permeability of TPS is considerably decreased by the incorporation of at least 20 wt% CNCs. Reinforcement is determined mostly by the competitive interactions among starch, nanocellulose, and plasticizer molecules. The aging of the films is caused by the additional water uptake from the atmosphere and the retrogradation of starch.

## 1. Introduction

Starch is a semicrystalline biopolymer that is readily available, inexpensive, and biodegradable. Despite being one of the most promising biopolymers, granular starch shows limited processability that makes the use of plasticizers necessary. Granules are destroyed while applying plasticizers and specific conditions, resulting in thermoplastic starch (TPS) showing a hydrophilic character and inadequate mechanical properties [1,2,3]. Different plasticizers are applied, but glycerol and sorbitol are the most commonly used in TPS. The type and amount of plasticizer strongly depend on the processing methods, as well the required properties of the TPS products [4,5,6,7,8,9,10,11,12,13,14,15,16,17,18,19,20]. As water can also act as a plasticizer for TPS, the moisture content of the samples significantly influences the measured characteristics. This also explains why the properties of the composites studied in different publications are difficult to compare.

TPS is also often modified with different types of nanocellulose, such as cellulose nanofiber (CNF) and nanocrystalline (CNC) cellulose [21,22,23]. While nanofibers are isolated from wood by mechanical treatment, cellulose nanocrystals are usually extracted with sulfuric acidic hydrolysis [23,24,25]. Most often, the effect of nanocellulose on mechanical properties (tensile test or dynamic mechanical analysis), and especially on reinforcement, is studied, for which an increase in modulus without an increase in strength is sometimes considered sufficient [12]. However, in most cases, there was a real reinforcement, so the tensile strength also increased with the filler content [11,13,26,27], but the increase was not significant in all cases. When using CNCs, the effect also depended on the shape of the nanocrystals and the properties of the TPS matrix, which is influenced by the type and amount of plasticizer. Occasionally, it has been found that strength has a maximum as a function of the cellulose content tested, which is explained by aggregation at higher concentrations [9,18,26,27].

In most cases, CNCs are found to increase thermal stability [18,25,26,28], but there is also an example of the opposite effect (i.e., there is an exception [9]). Water absorption and WVP, which are also favorably affected by nanocellulose intake, are also frequently studied [13,15,28]. Light transmission, on the other hand, is generally reduced by nanocellulose [16,18,26], although a study reports that 1% NC improved light transmission [17]. DMTA studies show that CNCs increase Tg, which is explained by the strong starch–CNC interaction [15,26]. However, this could also be caused by the CNC–glycerol interaction (less emollient effect). The strong cellulose–starch relationship is inferred from FTIR studies.

Retrogradation is the recrystallization process that can take place during the storage of water–polyol plasticized starch [29,30]. As a result, numerous properties such as opacity, hardness, deformability, dimensional stability, etc., change, having an effect on the final products in terms of shelf-life and overall quality. Some publications, including XRD, DSC, and rheological measurements, have also shown that fibrous cellulose and CNCs affect retrogradation. In the case of fibrous cellulose, this is explained by the lower/more moderate water uptake of the high crystallinity cellulose, which results in lower starch chain mobility [13]. In another publication, the authors explain the decreased post-crystallization of amylopectin by a strong starch–CNC interaction [31].

The large number of results published in the literature clearly indicates the attention on TPS–cellulose composites and the importance of the composites thus produced. However, despite the numerous results, many issues are still unclear, as the findings are often contradictory. In addition, interactions are rarely analyzed quantitatively, as the studied composition range is narrow, and the type and concentration of the plasticizer are not varied. In our research, starch was plasticized with glycerol or sorbitol at a 30 or 40% concentration, and then CNCs were applied as reinforcement in a wide composition range, up to 50 wt% in TPS. The mechanical and water vapor barrier properties, as well as the aging of TPS–CNC films, were characterized and investigated, with a particular focus on the structure and interactions developing in the composites.

## 2. Materials and Methods

### 2.1. Materials

The corn starch used in the experiments was supplied by Hungrana Ltd. (Szabadegyháza, Hungary). Glycerol with 0.5% water content, sorbitol, and sulfuric acid (96%) were obtained from Sigma-Aldrich Ltd. (Budapest, Hungary). Glycerol was used without further purification or drying. Bleached cotton, which can be considered pure (100%) cellulose, served as a cellulose source for the production of CNCs. Bleached cotton plain-weave fabric with a weight per unit area of 110 g m^−2^ was kindly provided by Pannon-Flax Linen Weaving Co. (Győr, Hungary) and used without any further wet treatment.

### 2.2. Sample Preparation

TPS solution was prepared as follows: native starch (6 g) was dispersed in 200 mL distilled water containing 30 or 40% plasticizer (such as glycerol or sorbitol). In order to gelatinize the corn starch particles, the suspension was continuously stirred with a magnetic stirrer at 80 °C for 30 min. For CNC production, the cotton fabric was ground in a Mixer Mill MM400 (Retsch GmBH, Haan, Germany), and the powder (10.0 g) was hydrolyzed with 64 wt% sulfuric acid at 45 °C for 25 min. The acid to cotton powder ratio was 8.75 mL/g. Subsequent to the post-treatments (washing, centrifugation, and dialysis), the total volume of the stock suspensions was subjected to ultrasonication for 10 min by using an ultrasonic horn-type reactor (Vibra-Cell VC505, Sonics & Materials, Newtown, CT, USA) at 60% amplitude with a driving frequency of 20 kHz [32]. The yield of CNCs calculated as a percentage of the initial weight of cotton powder was about 43%. The final aqueous suspensions contained about 4% cellulose nanocrystals by weight. The average length and width of the rod-like cellulose nanocrystals obtained from bleached cotton were 68 ± 5 nm and 8 ± 0 nm, respectively. TEM images of CNC suspensions showed a parallel arrangement of needle-shaped nanocrystals, leading to the formation of aggregates [24].

The CNC suspension was added in different percentages (0, 5, 10, 20, 50%) on the starch basis to the cold aqueous gelatinized starch, and the mixture was also ultrasonicated for 30 s, as described above. Rectangular films with a thickness of about 70 μm were cast from the TPS–CNC suspensions on the surface of a polypropylene plastic sheet and allowed to evaporate at room temperature for about 2 days. The detached films were stored and tested at 23 °C and 55% relative humidity in a conditioning room. Storage time was 1 week, 1 month, and 3 months. In some of the experiments, only selected compositions of TPS–CNC films were investigated because of the wide composition range studied in the research.

### 2.3. Characterization

For measuring the transmittance of films, a Unicam UV 500 (Budapest, Hungary) spectrophotometer was used in a wavelength range of 190–900 nm, and the transmittance values at 600 nm were compared and evaluated.

A Hunter Lab Color QUEST XE spectrophotometer (Reston, VA, USA) was used in total transmittance mode for measuring the Haze index of TPS–CNC films. Haze is defined as the percentage of transmitted light that, while passing through a specimen, deviates from the incident beam by more than 2.5° from the normal incident beam [33]. The values were determined in triplicate.

Morphology of the films was characterized by scanning electron microscopy (SEM) using JEOL JSM 6380 LA equipment (Tokyo, Japan). SEM micrographs were taken of the fracture surface of films, which were frozen in liquid nitrogen and subsequently broken.

The films were examined by polarized optical microscopy (POM) using a Zeiss Axioskop optical microscope (Jena, Germany) in which a λ-plate located diagonally between the crossed polarizers was used. The microscope was equipped with a Leica DFC 320 digital camera (Wetzlar, Germany).

Mechanical properties were determined using an Instron 5566 tensile tester (Norwood, MA, USA) equipped with a 500 N load cell. At least ten specimens with a size of 7 × 50 mm were cut from each of the films in different series. They were tested at a 10 mm/min cross-head speed and with a 20 mm span length.

Water vapor permeability (*WVP*) of the TPS–CNS films with a CNC content of 0, 5, 20, and 50% were determined. The film under test was sealed on the mouth of the jar containing distilled water that provides the 100% RH condition. The test jar was kept in a desiccator with an RH of 50% at 25 °C. The desired relative humidity was achieved with saturated solution of magnesium nitrate. The sealed jar was periodically removed and weighted. The decrease in weight of water in the jar was used to calculate the *WVP* of the sample. The following equation was used for calculation:

(1) $$WVP=\frac{\Delta m\cdot x}{A\cdot \Delta t\cdot \Delta p}$$

where *x* is the thickness of the film, *A* is the test area, ∆*m/*∆*t* is the slope of the straight line (weight loss per unit time), and ∆*p* is the difference between water vapor partial pressure inside and outside the cup.

The crystalline structure of TPS was studied by X-ray diffraction (XRD) using Philips PW1830/PW1050 equipment (Amsterdam, Holland) with Cu Kα radiation at 40 kV and 35 mA.

### 2.4. Graphical and Statistical Analysis

When data were plotted against CNC content, nonlinear curves were drawn by hand, and their only purpose is to show the tendency of measured data. Linear curves were fitted with the method of least squares.

The correlation coefficient of transmittance and haziness was calculated, and a *t*-test was performed to determine its statistical significance. The reinforcing effect of CNCs was statistically analyzed with a simple linear model. The continuous predictor was the CNC content, while the nominal factors were the type and amount of the plasticizer.

The level of significance was set at 0.05; thus, a factor was considered to be significant in the case of at *p* < 0.05. Calculations were carried out by means of Statistica software (Version 13.3, TIBCO Software, Palo Alto, CA, USA).

## 3. Results

### 3.1. Structure

**Figure 1:**
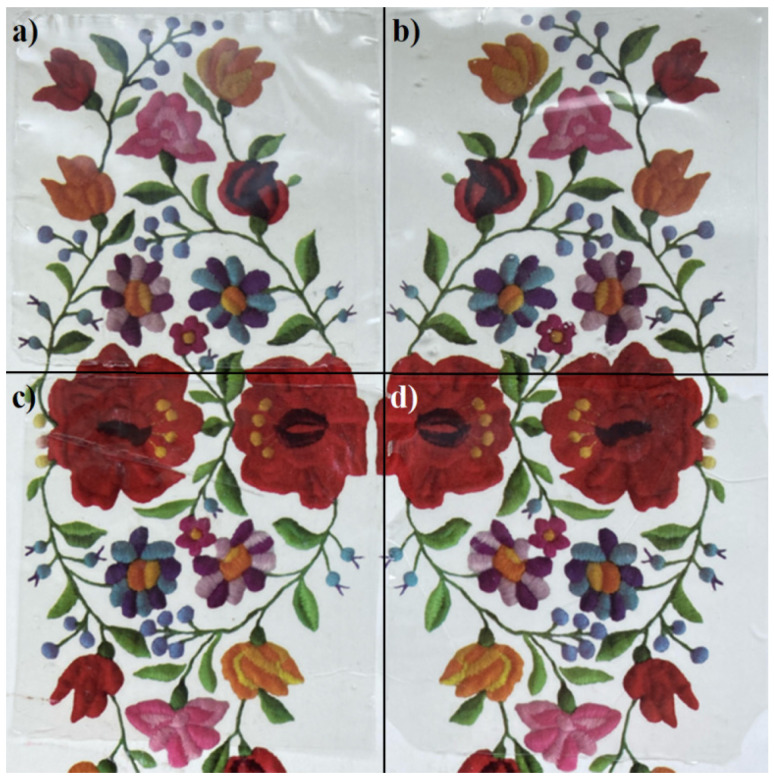
Transparent TPS–CNC films with different compositions: (**a**) 30% sorbitol and 50 wt% CNCs; (**b**) 40% sorbitol and 50 wt% CNCs; (**c**) 30% glycerol and 20 wt% CNCs; (**d**) 40% glycerol and 20 wt% CNCs.

**Figure 2:**
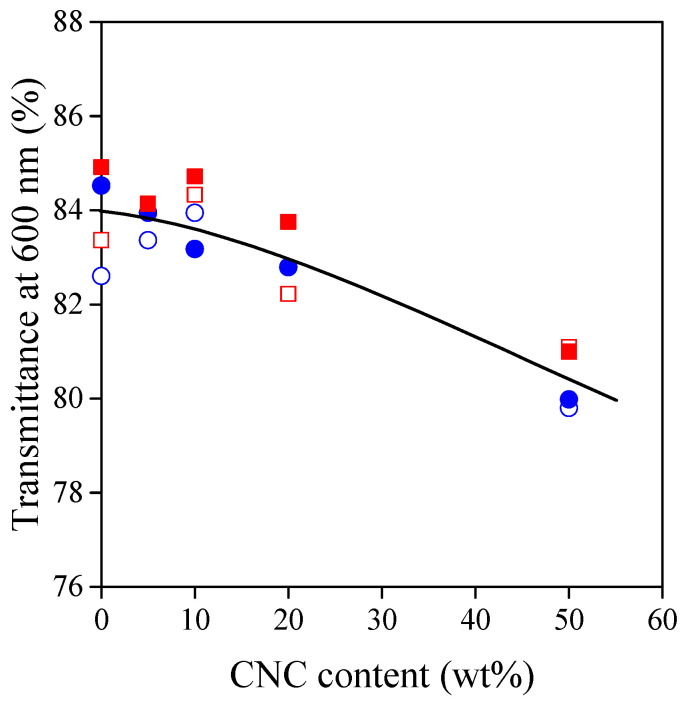
Transmittance of TPS–CNC composites at 600 nm as a function of CNC content. Symbols: (○) 30% glycerol; (●) 40% glycerol; (☐) 30% sorbitol; (■) 40% sorbitol.

In several packaging applications, transparency is a required property. As demonstrated in Figure 1, film casting resulted in smooth and transparent materials. The transparency of the films was characterized quantitatively by their visible spectrum and their Haze index. In the visible spectra of each composition, transmittance values at 600 nm were selected for comparison. Figure 2 shows that the transparency of the composites decreases with increasing CNC content. From a practical point of view, this decreasing tendency is negligible; thus, human eyes can hardly make any difference between the composites.

**Figure 3:**
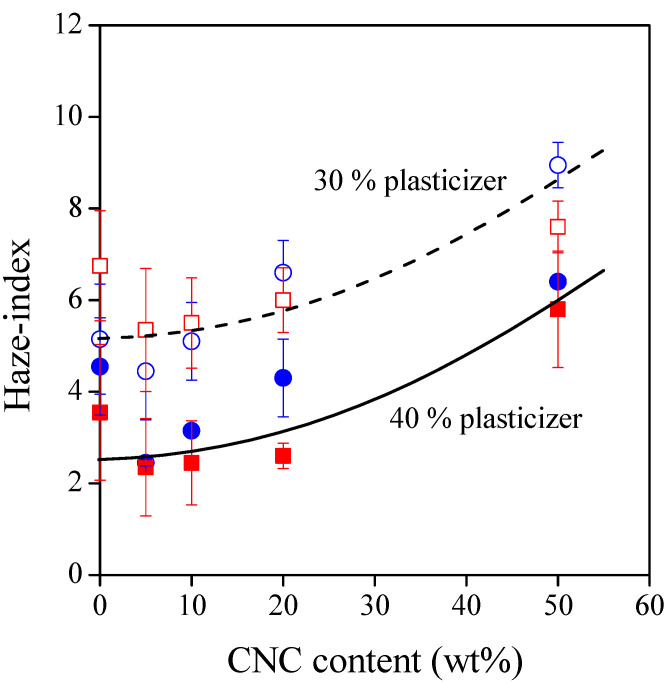
Haze index of TPS–CNC composites at 600 nm as a function of CNC content. Symbols: (○) 30% glycerol; (●) 40% glycerol; (☐) 30% sorbitol; (■) 40% sorbitol.

The lower transmittance of TPS–CNC composites may originate from increased absorbance or scattering, respectively. This latter is evidenced in Figure 3, in which Haze-indices follow the opposite tendency of transmittance. Furthermore, statistical analysis showed that the correlation between transmittance and haziness is extremely significant (*p* = 3.39 ×10^–4^). At any CNC content, the Haze index of the composites is determined mostly by the amount of the plasticizer. Higher plasticizer content results in less hazy materials due to the more homogeneous network of TPS and to the more homogeneous distribution of CNC particles.

In Figure 3, the increasing gradient of haziness can be related to the aggregation of CNCs. The aggregation ability of cotton nanocrystals was discussed in detail in our previous study [24]; therefore, we provide only a brief summary in this paper. Based on transmission electron microscopic images, cotton CNCs consist of rod-like particles with an average length of 68 nm and an average width of 8 nm. However, laser diffraction measurements showed that even at low concentrations, these nanocrystals form micron-sized particles in aqueous medium.

**Figure 4:**
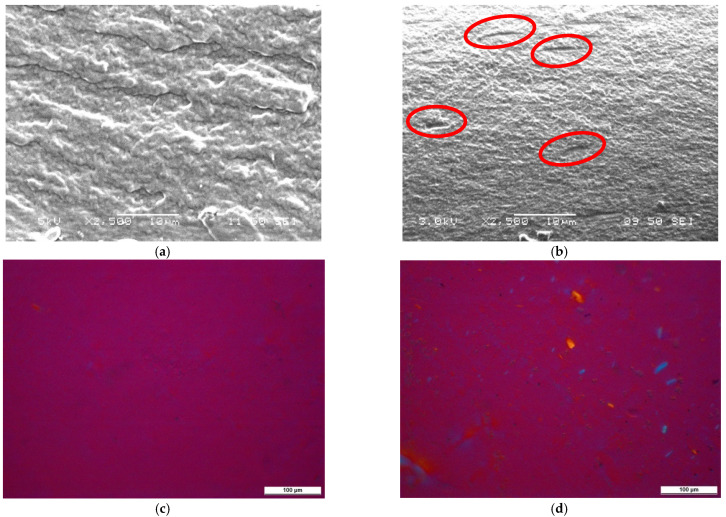
SEM images of cryogenic fracture surfaces and POM images of composite films: (**a**) SEM micrograph of TPS; (**b**) SEM micrograph of TPS with 20% CNCs; (**c**) POM micrograph of TPS; (**d**) POM micrograph of TPS with 20% CNCs. Plasticizer: 40% glycerol.

The formation of CNC aggregates in TPS is corroborated by microscopic images. Figure 4 shows the SEM and POM micrographs of glycerol-plasticized TPS and its composite with 20 wt% CNCs, respectively. Several micron-sized cavities appeared on the cryogenic fracture surface of TPS–CNC composite (Figure 4a), while they cannot be observed on the fracture surface of neat TPS (Figure 4c). Such cavities are the imprints of nanocrystal aggregates that remain after the debonding of the TPS matrix and the CNC particles. The aggregation of CNCs is evidenced also by the POM images. In Figure 4c,d, TPS is nonbirefringent and has a purple color, while in Figure 4d, cellulose is birefringent and has a yellow or blue color.

### 3.2. Properties

**Figure 5:**
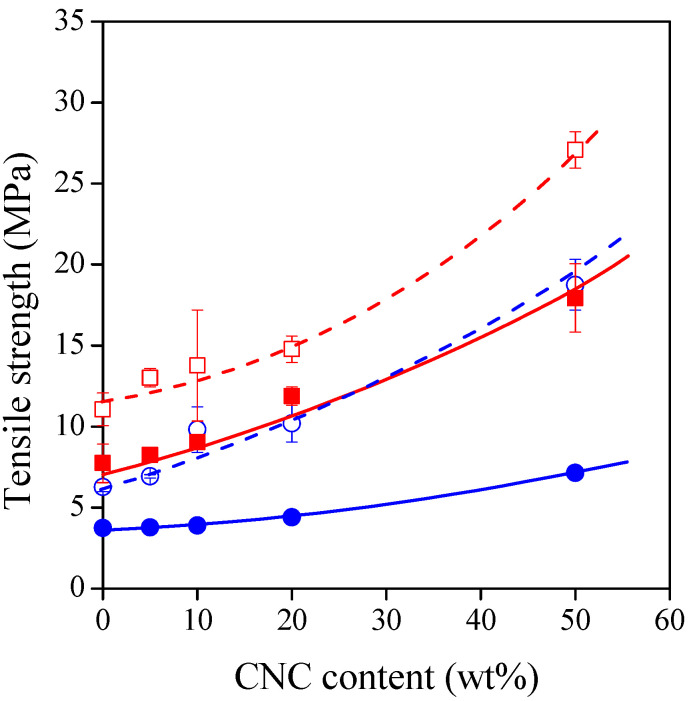
Tensile strength of TPS–CNC composites as a function of CNC content. Symbols: (○) 30% glycerol; (●) 40% glycerol; (☐) 30% sorbitol; (■) 40% sorbitol.

The inferior mechanical properties of TPS represent the largest obstacle to practical application in many cases. Fiber reinforcement is a simple and efficient way to increase the strength of TPS while maintaining acceptable toughness. Although CNCs form micron-sized aggregates, their reinforcing effect is still remarkable (Figure 5). The increase in tensile strength is determined significantly by the type and amount of plasticizer. The least significant effect was observed for the composites prepared with 40% glycerol, as strength was increased from 3.7 Mpa only to 7.1 Mpa. The most significant effect was revealed for the composites containing 30% sorbitol, as strength was enhanced from 11.1 Mpa to 27.1 Mpa. This observation demonstrates well the role of interactions and structure in the determination of strength. These factors will be studied in detail in the next section.

**Figure 6:**
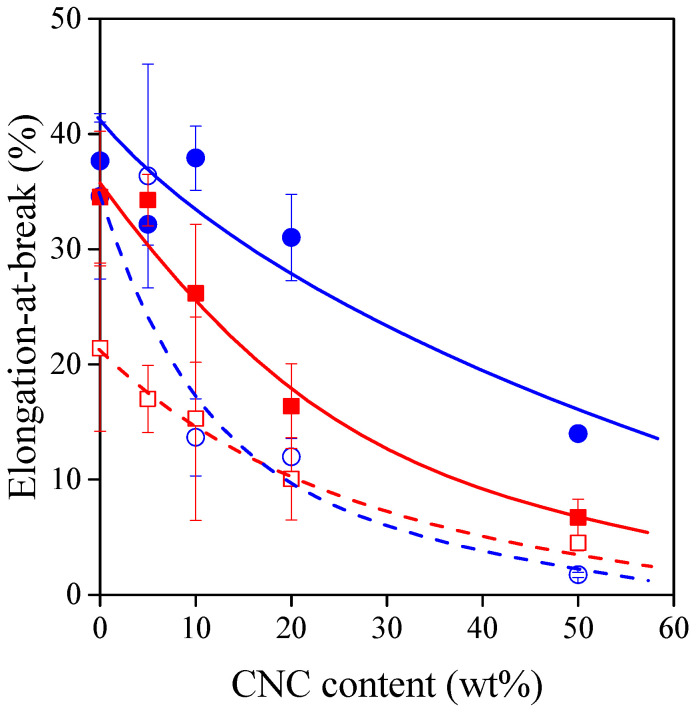
Elongation-at-break of TPS–CNC composites as a function of CNC content. Symbols: (○) 30% glycerol; (●) 40% glycerol; (☐) 30% sorbitol; (■) 40% sorbitol.

The deformability of the materials studied is represented by their elongation-at-break value as a function of CNC content in Figure 6. The incorporation of rigid nanocrystals decreases the deformability of the composites. The tendencies are affected by the type and amount of plasticizer. The decrease in deformability is the least significant for the composites containing 40% glycerol. However, as seen in Figure 5, this composite series has the lowest strength. This means that high deformability and high strength exclude each other for the TPS–CNC composites studied, but they can be balanced according to the requirements of the selected packaging application.

**Figure 7:**
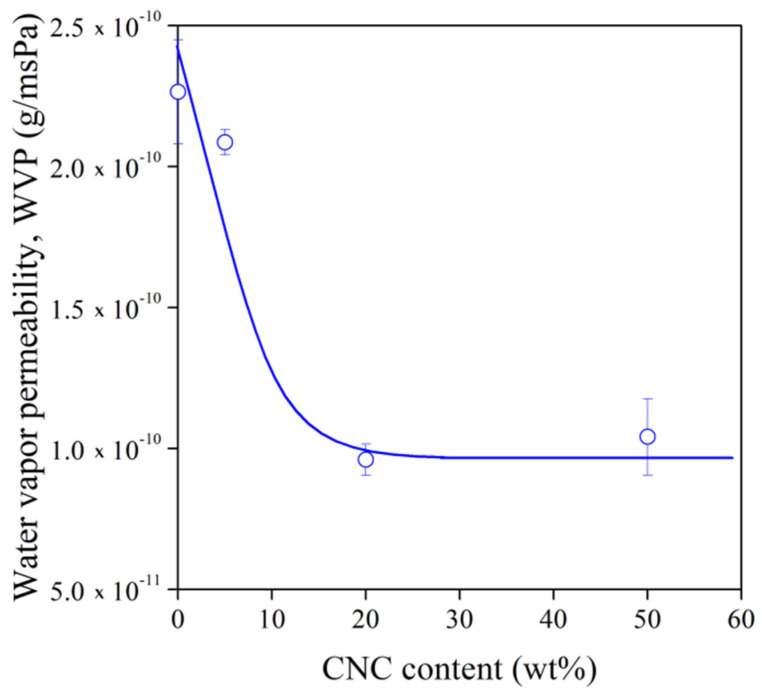
Water vapor permeability of TPS–CNC composites with 30% glycerol as a function of CNC content.

For packaging materials, gas and vapor permeability is often an important factor. TPS films are relatively good barriers against oxygen but poor against water vapor [34,35]. The incorporation of fillers or fibers in a polymer matrix can increase the diffusion length of water vapor resulting in hindered permeation [13,15,28,34,35]. The efficiency of this approach is demonstrated in Figure 7. For the composite series prepared with 30% glycerol, WVP decreases steeply until 20 wt% CNC content, and it becomes almost constant between 20 and 50 wt%.

The following formula was used to characterize quantitatively the improvement of water vapor barrier:

(2) $$eff_{WVP}=100\%\frac{WVP_{0}-WVP_{m}}{WVP_{0}}$$

where *ef f_WVP_* is the effect of fiber incorporation on *WVP*, while the indices 0 and m denote the neat TPS and its composite with the highest fiber content, respectively. In Table 1, our results are compared to the data of cellulose microfiber-reinforced TPS composites. As other composites were prepared only up to 1 or 10 wt% fiber content, we also estimated the *ef f_WVP_* value at 1 and 10 wt% for our TPS–CNC composites from the fitted curve in Figure 7. The effect of CNCs was smaller in our films than in the composites of Ilyas et al. [28], which can be related to the distinct sample preparation process and to the different plasticizer systems. Nevertheless, despite the aggregation of nanocellulose, CNCs hinder water diffusion more significantly than cellulose microfibers.

### 3.3. Reinforcement

As previously shown, the tensile properties of TPS–CNC films can be tailored by the type and amount of the plasticizer. These factors determine directly the interactions in all phases, i.e., in the TPS matrix, the CNC aggregates, and the starch–cellulose interphase as well. Interactions can be analyzed quantitatively from the composition dependence of tensile strength by using a simple model [36]:

(3) $$\sigma_{T}\frac{1+2.5\varphi }{1-\varphi }=\sigma_{Tred}=\sigma_{T0}\;exp\left(B\varphi \right)$$

where *σ_T_* and *σ_T_*_0_ are the true tensile strength of the TPS–CNC composite and the neat TPS, respectively, *φ* is the volume fraction of CNCs that was estimated from the weight fraction by using the density of the components, *σ_Tred_* is the reduced tensile strength, and *B* is the relative load-bearing capacity of TPS and CNCs. Parameter *B* is the component of the model that can be related to interactions, and it can be expressed as [37]

(4) $$B=\left(1+A_{f}\rho_{f}\ell \right)ln\left(\frac{\sigma_{Ti}}{\sigma_{T0}}\right)$$

where *A_f_* and *ρ_f_* are the specific surface area and density of the CNC particles, while *ℓ* and *σ_Ti_* are the thickness and the strength of the starch–cellulose interphase, respectively. The value of *B* can be easily determined by taking the logarithm of both sides of Equation (3):

(5) $$ln\sigma_{Tred}=ln\left(\sigma_{T}\frac{1+2.5\varphi }{1-\varphi }\right)=ln\sigma_{T0}+B\varphi$$

**Figure 8:**
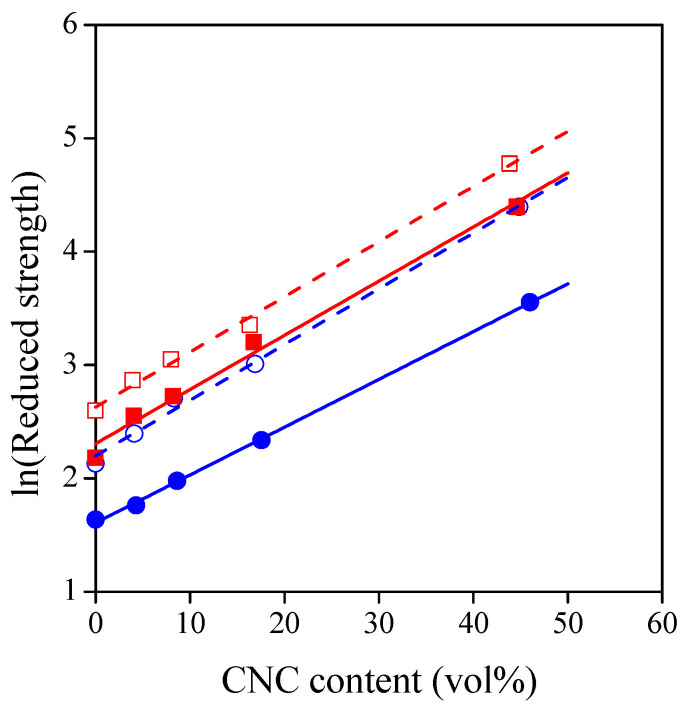
Reduced strength of TPS–CNC composites as a function of CNC content. Reduced strength is defined in Equation (3). Symbols: (○) 30% glycerol; (●) 40% glycerol; (☐) 30% sorbitol; (■) 40% sorbitol.

Equation (5) is the equation of a line, and its slope is parameter *B*. The tensile strength of the TPS–CNC composite series is plotted this way in Figure 8, while the fitting results are summarized in Table 2. For the composite series studied, only the plasticizer content has a significant effect on the *B* values (*p* = 0.005138); thus, higher plasticizer content results in a lower *B* parameter. However, this does not necessarily mean that only the concentration of plasticizer determines the interactions. The strength of the matrix has a strong effect on the value of *B* as well [38,39,40]. To avoid this effect, parameter *B* was multiplied by the natural logarithm of the true tensile strength of the TPS [40], and these values (*B*∙*lnσ_T_*_0_) are also placed in Table 2. The effect of plasticizer type is revealed by the *B∙lnσ_T_*_0_ values, and it seems that the reinforcing effect of CNCs is higher in the sorbitol-plasticized systems at a certain plasticizer content.

As discussed in Section 3.1, increasing the amount of plasticizer from 30% to 40% resulted in a more homogeneous distribution of CNC aggregates in the TPS matrix. This means an increased interfacial area between starch and cellulose and a higher reinforcing effect based on Equation (4). Nevertheless, parameter *B* was lower at higher plasticizer content; therefore, an additional factor overcompensated for the effect of structure. According to several previous papers [1,4,41,42], competitive interactions may develop among starch, the plasticizer molecules, and the filler particles, which determines the extent of reinforcement in TPS-based composites. Increasing the amount of plasticizer from 30% to 40% can hinder the interaction between starch and CNCs. As a result, stress transfer between TPS and CNC may deteriorate [1,4,41,42]. Furthermore, plasticizer molecules can migrate into the CNC aggregates; therefore, their inherent strength may decrease as well [11].

Plasticizer type has a significant effect on the strength of TPS. When plasticizers are added to starch on a mass basis, glycerol is a more efficient plasticizer than sorbitol; thus, a higher number of polymer–polymer interactions are changed to polymer–plasticizer interactions. As a consequence, sorbitol-plasticized films have higher strength than glycerol-plasticized films [43,44,45], which is in agreement with our results at zero CNC content in Figure 5.

Plasticizer molecules can migrate into the CNC aggregates, and they can change the interactions between the nanocellulose crystals; therefore, their inherent strength is determined also by the effect of plasticizer type. In our previous study, it was shown that neat CNC films had a maximum strength at 15% glycerol or sorbitol content on the basis of CNCs, and above this concentration, sorbitol-plasticized films had a significantly higher strength [24]. As the molecular character is very similar for starch and cellulose, we can assume that the strength of the starch–cellulose interphase is also higher in the case of sorbitol. Based on Equation (4), this can explain the higher reinforcing effect of CNCs in sorbitol-plasticized TPS.

In Table 2, the fitting results of TPS–cellulose composites are summarized for comparison. The *B∙lnσ_T_*_0_ values show that CNCs have an acceptable reinforcing effect in TPS compared to cellulose microfibers. Nevertheless, the potential of CNC reinforcement could be only partially exploited in our composites as the nanocrystals tend to aggregate at a high fiber content.

### 3.4. Aging of Composites

**Figure 9:**
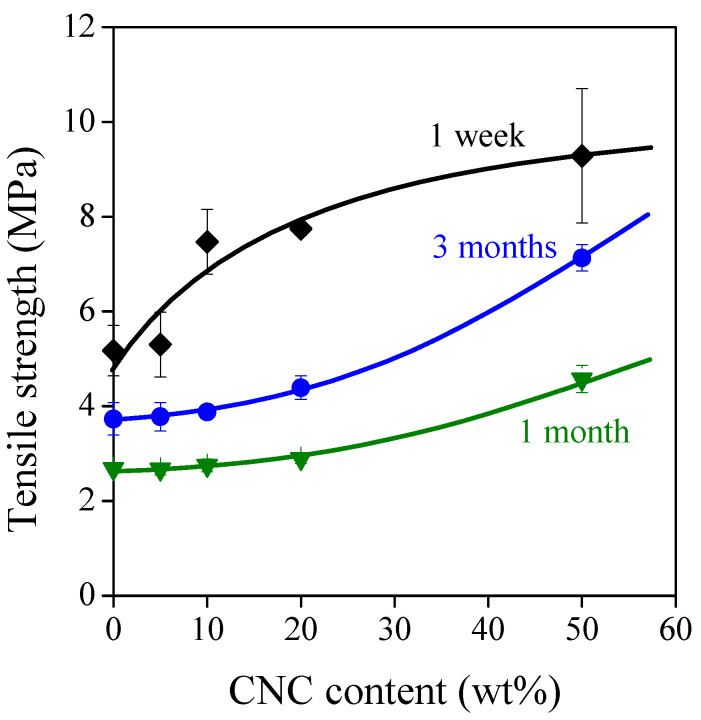
Effect of aging time on the tensile strength of TPS–CNC composites. Plasticizer: 40% glycerol. Symbols: (♦) 1 week; (▼) 1 month; (●) 3 months.

The properties of TPS alter with time since plasticization enables the segmental motion of amylose and linear parts of amylopectin molecules, triggering a recrystallization process called retrogradation [29,30]. Mechanical properties are extremely sensitive to the changes in the crystalline structure; therefore, the aging of TPS composites was characterized by their tensile strength values after 1 week, 1 month, and 3 months (Figure 9). Previous studies showed that the retrogradation of TPS results in an increase in tensile strength due to the increased crystalline ratio [7,13]. Nevertheless, Figure 9 shows that the strength of TPS and TPS–CNC composites has a minimum value after 1 month, and it does not gain back its original value even after 3 months. This means that there is another process competing with retrogradation.

**Figure 10:**
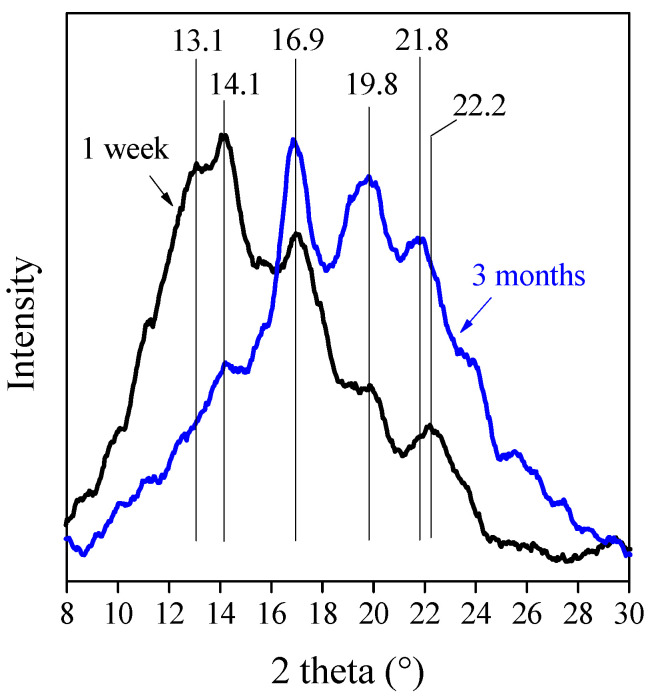
XRD diffractograms of TPS after 1 week and 3 months. Plasticizer: 40% glycerol.

The changes in the crystalline structure of TPS are demonstrated by XRD diffractograms in Figure 10. The two theta peaks at 13.1° and 14.1° represent the V_a_-type crystals, while the peaks at 16.9°, 19.8°, 21.8°, and 22.2° appear due to the presence of V_h_-type crystals [46,47]. The diffractograms prove unambiguously that V_a_-type crystals transform into V_h_-type crystals during aging. According to Zobel et al. [48], the amylase helices of V_a_ lattice are more contracted and they contain less water compared to V_h_ lattice. This implies that the transformation of V_a_-type crystals originates from the hydration of TPS, which is caused by the additional water uptake from the atmosphere. Based on the results of Dai et al. [49], this recrystallization process is reversible; thus, the V_h_-type crystals can transform back to V_a_-type crystals in dehydration conditions.

Figure 10 corroborates that the aging of TPS and TPS–CNC composites is determined by the competition of water uptake from the atmosphere and the retrogradation of starch. The increasing amount of water has a plasticizing effect; therefore, it decreases strength. In the meantime, retrogradation increases strength due to the increasing amount of crystalline phase. As the diffusion of water is faster than recrystallization, strength goes through a minimum during aging.

## 4. Conclusions

The inferior properties of TPS films, such as high water vapor permeability and low strength, were improved by the incorporation of CNCs, while their transparency decreased negligibly. In these composites, structure and interactions were modified with glycerol or sorbitol as a plasticizer at a 30 or 40% concentration on a starch basis. Additionally, CNC content varied in a wide range, up to 50 wt% on the basis of starch. In the composite films, micron-sized CNC aggregates were observed. Nevertheless, CNCs improved the water vapor barrier more significantly than some cellulose microfibers. The reinforcing effect of CNCs was higher in the composites prepared with 30% plasticizer, as the formation of starch–cellulose interactions may be less hindered at lower plasticizer concentration, and the inherent strength of CNC aggregates may be higher. Additionally, the extent of reinforcement was higher in sorbitol-plasticized films than glycerol-plasticized films, which can be explained by the higher strength of the interphase and the CNC particles, respectively. Although the reinforcing effect of CNCs could be only partially exploited due to aggregation, it was somewhat higher compared to some cellulose microfibers. During the aging of the composites, strength went through a minimum value, which originated from the competition of water uptake from the atmosphere and the retrogradation of starch. Strength was decreased by the plasticizing effect of water, while it was increased by the retrogradation of starch.

## Figures and Tables

**Table 1:** Comparison of water vapor barrier improvement in TPS–cellulose films.

Fiber	Plasticizer	Plasticizer Content (%)	Fiber Content, (wt%)	*eff_WVP_* (%)	Reference
CNC	Glycerol	30	50	60	This work
CNC	Glycerol	30	10	47	This work
CNC	Glycerol	30	1	6.7	This work
CNC	1:1 glycerol–sorbitol	30	1	18.5	[28]
Cellulose from rice husk	Glycerol	30	10	4.3	[34]
Microcrystalline cellulose	Glycerol	30	10	20	[35]

**Table 2:** Reinforcement in TPS–cellulose composites.

Fiber	Plasticizer	Plasticizer Content (%)	Parameter *B*	*B*∙ln*σ_T0_*	Goodness of Fit, R^2^ *	Reference
CNC	sorbitol	30	4.87	12.65	0.9968	this work
CNC	sorbitol	40	4.12	9.64	0.9993	this work
CNC	glycerol	30	4.92	10.49	0.9952	this work
CNC	glycerol	40	4.22	6.90	0.9993	this work
Cellulose from rice husk	glycerol	30	4.05	8.68	0.8560	[34]
Microcrystalline cellulose	glycerol	30	2.73	1.95	0.9760	[35]

* Determination coefficient indicating the goodness of the fit.

## Data Availability

The data presented in this study are available on request from the corresponding author.
